# Evolution of carbapenem resistance in *klebsiella pneumoniae* and *escherichia coli* carrying *bla*_NDM−1_ gene: imipenem exposure results in sustained resistance memory of strains in vitro

**DOI:** 10.1186/s12941-023-00598-8

**Published:** 2023-06-12

**Authors:** Qiong Zhao, Longhua Sha, Zhaomeng Wu, Lixue Meng, Feixiang Yang, Lingling Wu, Chunfang Yu, Hua Zhang, Jingdan Yu, Zhixiong Jin

**Affiliations:** 1 Department of Microbiology, School of Basic Medical Sciences, Hubei University of Medicine, Hubei, 442000 Shiyan China; 2 Department of Clinical Laboratory, Sinopharm Dongfeng General Hospital, Hubei University of Medicine, Hubei, Shiyan, 442008 China; 3 Laboratory Medicine, Wuhan Asia General Hospital, Hubei, Wuhan, 430050 China; 4 Hubei Key Laboratory of Wudang Local Chinese Medicine Research, Hubei University of Medicine, Hubei, 442000 Shiyan China

**Keywords:** *Klebsiella pneumoniae*, *Escherichia coli*, *β*-lactamase, *bla*_NDM−1_, Imipenem, Drug resistance

## Abstract

**Background:**

Antibiotics exert an outstanding selective pressure on bacteria, forcing their chromosomal gene mutations and drug resistance genes to spread. The objective of this study is to evaluate the expression of the New Delhi Metallo-β-Lactamase-1 gene (*bla*_NDM−1_) in the clinical isolate (*Klebsiella pneumoniae* TH-P12158), transformant strains *Escherichia coli* BL21 (DE3)-*bla*_NDM−1_, and *Escherichia coli* DH5α- *bla*_NDM−1_ when exposed to imipenem.

**Methods:**

*β*-Lactamase genes (*bla*_SHV_, *bla*_TEM−1_, *bla*_CTX−M−9_, *bla*_IMP_, *bla*_NDM−1_, *bla*_KPC_, *bla*_OXA_, *bla*_GES_, and *bla*_DHA_) from randomly selected carbapenems-sensitive *K.pneumoniae* (n = 20) and *E.coli* (n = 20) strains were amplified by PCR. The recombinant plasmid of pET-28a harboring *bla*_NDM−1_ was transformed into *E.coli* BL21 (DE3) and *E.coli* DH5α by electroporation. The resistance phenotype and higher *bla*_NDM−1_ expression in *K.pneumoniae* TH-P12158, transformant *E.coli* BL21 (DE3)-*bla*_NDM−1_, and *E.coli* DH5α-*bla*_NDM−1_ were observed when exposed to imipenem with grade increasing, decreasing, and canceling doses, respectively.

**Results:**

After being exposed to different doses of imipenem, the minimum inhibitory concentration (MIC) and the minimum bactericidal concentration (MBC) of antimicrobial drugs and *bla*_NDM−1_ expression of strains increased, which was positively correlated with doses of imipenem. On the contrary, with the decrease or cancellation of imipenem doses, the *bla*_NDM−1_ expression was deteriorated, while the MIC and MBC values remained relatively stable. These results demonstrated that low doses of imipenem (˂MIC) could press *bla*_NDM−1_ positive strains producing stable drug resistance memory and altered *bla*_NDM−1_ expression.

**Conclusions:**

Low doses of imipenem could press *bla*_NDM−1_ positive strains producing sustained resistance memory and altered *bla*_NDM−1_ expression. In particular, the positive correlation between the resistance genes expression and antibiotics exposure shows promising guiding significance for clinical medication.

**Supplementary Information:**

The online version contains supplementary material available at 10.1186/s12941-023-00598-8.

## Background

The gram-negative bacteria family *Enterobacteriaceae* has become the main source of both community and hospital-acquired infections that range from abscesses to blood infections, intra-abdominal infections, meningitis, pneumonia, and urinary tract infections [1]. Furthermore, the continuous emergence of drug-resistant *Enterobacteriaceae* (DRE) leads to increased morbidity, mortality, and healthcare costs [2]. The typical effect of DRE is the inactivation of *β*-lactams by *β*-lactamases such as extended-spectrum *β*-lactamases (ESBLs), AmpC cephalosporinases, and carbapenemases [3]. According to Ambler classification (similarity of amino acid sequences), *β*-lactamases are divided into four categories of class A, B, C, and D. Key enzyme families includes TEM, SHV, CTX-M, and KPC (class A); IMP, VIM, SPM, GIM, SIM, and NDM (class B); CMY, DHA, and ADC (class C); Class D enzymes are all termed OXA [4–10]. Class A, C, and D enzymes contain a serine residue at the active site of *β*-lactamase, while class B enzymes (Metallo-*β*-Lactamases, MBLs) contain one or two zinc ions at the active site. The genes encoding MBLs are located in both chromosome and plasmids, the primary structure of the enzyme is highly variable, and the homology of the amino acid sequence is less than 23% [11, 12]. In 2009, Timothy R. Walsh discovered a new type of MBLs, New Delhi Metallo-β-Lactamases-1 (NDM-1), hydrolyzes *β*-lactams (including carbapenems), which greatly damage antibacterial chemotherapy based *β*-lactam [13]. Nowadays, the *bla*_NDM−1_ encoding NDM has already disseminated worldwide and has been detected in *Enterobacteriaceae*, including *Klebsiella pneumoniae*, *Escherichia coli*, *Acinetobacter baumannii*, *Pseudomonas aeruginosa, Enterobacter cloacae*, and *Raoultella ornithinolytica* [13–15].

*Enterobacteriaceae* is able to capture, accumulate and transmit resistance genes through the migration of gene elements (plasmids, insertion sequences, transposons, and integrons) within and between species [16, 17]. *bla*_TEM,_*bla*_SHV_, and *bla*_CTX−M_ have become the main epidemic trend, the subtypes and variants of these gene families are developing and spreading geographically and in a variety of bacterial species [8], *E.coli* and *K.pneumoniae* are important bacteria for accumulating these resistance genes. The plasmids carrying *bla*_NDM−1_ isolated *Enterobacteriaceae* also coexist with other drug resistance genes (*bla*_NDM−5_, *bla*_OXA23_, *bla*_OXA52_, *armA*, *bla*_TEM−1_, and *bla*_CTX−M−9_), which confer resistance to different classes of antibiotics [14, 18, 19]. Moreover, these plasmids carrying multiple drug-resistance genes can be exchanged by conjugation within different enterobacterial species [14].

Carbapenems are the mainstay antimicrobial agents for treating severe infection for their strong antibacterial activity and comprehensive antibacterial spectrum against gram-positive and gram-negative bacteria [20]. *β*-lactamase inhibitors cannot prevent the hydrolysis of carbapenems induced by NDM-1. Facing the reality that *bla*_NDM−1_ bacteria spread worldwide and greatly weaken the application effect of antibiotics, researchers have paid too much attention to the mechanism of resistance (mutation and gene transfer) and coping strategies [21, 22], although little attention is paid to the evolution of drug resistance of carbapenemase producing strains. In this study, we conducted the laboratory evolution of *K.pneumoniae* and *E.coli* carrying *bla*_NDM−1_ gene exposed to imipenem in vitro, to provide theoretical support for clinical control of the transmission of *bla*_NDM−1_ and the infection of the strain carrying it.

## Methods

### Bacterial strains

A total of 102 strains of *K.pneumoniae* and 91 strains of *E.coli* were isolated from sputum, blood, urine, cerebrospinal fluid, and secretions of patients admitted at the University Affiliated Hospital in China from July to August 2020. The VITEK-2 compact automatic microbiological analysis system (bioMerieux, Marcy-l′Etoile, France) is used to detect the minimum inhibitory concentration (MIC) of clinically recommended antibiotics. At the same time, the sensitivity of carbapenems was verified by the disk diffusion method (K-B method) [23].

*E.coli* BL21 (DE3) and *E.coli* DH5α strains (Novagen, Darmstadt, Germany) were used as an expression cell of pET-28a (+)-*bla*_NDM−1_ plasmid in the laboratory.

### Molecular identification of ***β***-lactamase genes

All randomly selected carbapenems-sensitive *K.pneumoniae* (n = 20) and *E.coli*(n = 20) strains were identified by 16SrDNA sequence analysis. The ESBLs (*bla*_SHV_, *bla*_TEM−1_, and *bla*_CTX−M−9_), AmpC (*bla*_DHA_), and carbapenemase *(bla*_IMP_, *bla*_NDM−1_, *bla*_KPC_, *bla*_GES_, and *bla*_OXA_) resistance genes carried by these strains were amplified by polymerase chain reaction (PCR) as previously described [14]. Primers for PCR were shown in Table 1. Amplification system: 2×NovoStar Green PCR Mix 25µL, Upstream, downstream primer 1µL (10µmol/L), DNA template 1µL, ddH_2_O 22µL, total volume 50µL. Amplification conditions: pre-denaturation at 95 °C for 5 min, denaturation at 95 °C for 30s, annealing at 55 °C (*bla*_SHV_) or 49 °C (*bla*_TEM−1_) or 50 °C (*bla*_CTX−M−9_) or 56 °C (*bla*_IMP_) or 55 °C (*bla*_NDM−1_) or 52 °C (*bla*_KPC_) or 57 °C (*bla*_OXA_) or 56 °C (*bla*_GES_) or 50 °C (*bla*_DHA_) for 30s (Table 1), extension at 72 °C for 1 min, a total of 34 cycles, extension at 72 °C for 5 min. Amplified products were separated by 1% agarose gel electrophoresis (110 V, 30 min) and sequenced. The gene sequence was compared with the GenBank database to determine the genotype.

**Table 1:** The list of primers used for the amplification of *β*-lactamase genes

Gene	Primer sequence (5^'^→3^'^)	Gene length (bp)	Tm (℃)
*bla*_SHV_^a^	F:ATGCGTTATATTCGCCTGTG	843	55
	R:TTAGCGTTGCCAGTGCTC		
*bla*_TEM−1_^b^	F:AGTATTCAACATTTTCGTGT	860	49
	R:TAATCAGTGAGGCACCTATCTC		
*bla*_CTX−M−9_	F:CGTATTGGGAGTTTGAGATG	522	50
	R:GGTATTCAGCGTAGGTTC		
*bla*_IMP_	F:GCGTTTATGTTCATACTTCGTT	631	56
	R:GCTTCTAAATTTGCGTCACC		
*bla*_NDM−1_	F:ATGGAATTGCCCAATATTATGCAC	813	55
	R:TCAGCGCAGCTTGTCGGCCATGCG		
*bla*_KPC_	F:CGTTCTTGTCTCTCATGGCC	796	52
	R:CCTCGCTGTGCTTGTCATCC		
*bla*_OXA_	F:CTGGAATGAGAATAAGCAGCAA	545	57
	R:GTTCAACCCAACCGACCC		
*bla*_GES_	F:ATGCGCTTCATTCACGCAC	863	56
	R:CTATTTGTCCGTGCTCAGGA		
*bla*_DHA_	F:GTTGCCGTCTCCGTAAAG	925	50
	R:GAATCACAATCGCCACCT		

Primer sequences for amplifying group genes (a) or single gene subtypes (b)

### ***Bla***_NDM−1_-pET28a (+) plasmid

According to the literature, the *bla*_NDM−1_ gene was amplified by PCR and cloned into a pET-28a vector containing a 6×His tag.

Primers *bla*_NDM−1_ (Fwd-5′-GGATCCATGGAATTGCCCAATATTATGCA-3′ and Rev-5′-GTCG ACTCAGCGCAGCTTGTCGGCCAT-3′) were designed with BamH Ӏ & Sal Ӏ enzyme digestion sites added at both ends. The pCYNDM01 plasmid DNA (accession no. MK510953) was used as a template for PCR amplification of the *bla*_NDM−1_ gene (837 bp). Amplification reactions were performed in a total volume of 20µL (2×Taq PCR Green Mix 10µL, NDM-1-Fwd 0.5µL, NDM-1-Rev 0.5µL, DNA 1µL, and ddH_2_O 8µL). The mixture was heat denatured at 95 °C for 30s, annealed at 55 °C for 30s, extended at 68 °C for 2 min, and the reaction was carried out for 35 cycles. The PCR products were detected by 1% agarose gel electrophoresis (110 V, 27 min) and purified by PCR purification Kit (Qiagen).

5µL of PCR products (*bla*_NDM−1_) and pET28a (+) vector containing a 6×His tag used in a enzyme digestion reaction with BamH Ӏ 0.5µL, Sal Ӏ 0.5µL,10×Buffer 2µL, and ddH_2_O 8µL in a total volume of 20µL was incubated for 4 h at 37 °C. Then, the reaction system of 20µL (*bla*_NDM−1_ 6µL, pET28a (+) 2µL, T4 DNA ligase 1µL, and 10× Ligase Buffer 1µL) was connected overnight at 4 °C.

The recombinant plasmid was sequenced and transformed into *E.coli* BL21 (DE3) and *E.coli*DH5α as described previously [24]. After overnight incubation at 37 °C (200 rpm) in LB liquid medium, the positive expression strains *E.coli* BL21 (DE3)-*bla*_NDM−1_ and *E.coli* DH5α-*bla*_NDM−1_ were screened on LB solid medium containing 100 µg/mL kanamycin and 0.5 µg/mL imipenem.

### Imipenem exposure

According to the clinical guidance, a MIC of ≥ 4 µg/mL for imipenem is considered as carbapenems-resistant *Enterobacteriaceae* [25]. Different doses of imipenem were assessed for their effects on the drug resistance phenotype and genotype of clinical isolate *K.pneumoniae* TH-P12158 (carbapenem-sensitive strain carrying *bla*_NDM−1_ gene), *E.coli* BL21 (DE3)-*bla*_NDM−1_, and *E.coli* DH5α-*bla*_NDM−1_.

### Subculture growth

Taking OD_600_ of 1.5-2.0 (1.5 × 10^8^ CFU/mL) as the subculture growth standard of strains, *K.pneumoniae* TH-P12158, *E.coli* BL21 (DE3)-*bla*_NDM−1_, and *E.coli* DH5α-*bla*_NDM−1_ were incubated in Mueller-Hinton (MH) liquid medium containing 0.5×MIC imipenem at 37 °C for 200 rpm until the OD_600_ was 1.5-2.0. The same process was repeated until the OD_600_ value of the strain reached 1.5-2.0 within 11–12 h at the same imipenem concentration, and then the next higher imipenem concentration was exposed for subculture growth.

### MIC, MBC, and K-B method

Regardless of whether imipenem increased (4 µg/mL, 8 µg/mL and 12 µg/mL), decreased (12 µg/mL to 8 µg/mL, 8 µg/mL to 4 µg/mL, and 4 µg/mL to 0 µg/mL) or canceled (12 µg/mL to 0 µg/mL, 8 µg/mL to 0 µg/mL, and 4 µg/mL to 0 µg/mL), the MIC and the minimum bactericidal concentration (MBC) of seven antimicrobial drugs (imipenem, meropenem, cefuroxime, ceftazidime, cefoperazone sodium/sulbactam sodium, piperacillin sodium/tazobactam sodium, levofloxacin) against three strains exposed to imipenem were detected, respectively. Meanwhile, the K-B method was used to verify the sensitivity of three strains to imipenem.

The MIC was interpreted using CLSI M100 (in 2019). Antimicrobial drugs were diluted into Mueller-Hinton liquid medium at a final ratio of 1280 µg/mL, 640 µg/mL, 320 µg/mL, 160 µg/mL, 80 µg/mL, 40 µg/mL, 20 µg/mL, 10 µg/mL, 5 µg/mL, and 2.5 µg/mL. A volume of 90µL strain (1.5 × 10^8^ cfu/mL) was seeded in each well of 96-well plates containing 10µL antibacterial drugs and incubated for 16-24 h at 37 °C. The MIC of antibacterial drugs was evaluated by absorbance value at 600 nm. All experiments were repeated for 3 times.

100µL of bacterial solution from 96-well plates exhibiting complete bacteriostatic activity were incubated on MH solid medium without antibiotics at 37 °C for 48 h, and the minimum drug concentration without bacterial growth was denoted as the minimum bactericidal concentration (MBC). All experiments were repeated for three times.

A volume of 100µL three strains (1.5 × 10^8^ cfu/mL) were inoculated on MH solid medium, respectively. The disk containing imipenem of 10 µg was attached to the plate and incubated at 37 °C for 18-20 h. The diameter of the inhibition zone was considered to be the degree of drug resistance of strains exposed to imipenem. The experiment was repeated three times to calculate the mean and standard error.

### Quantitative real-time-PCR (qRT-PCR)

The qRT-PCR was used to detect the expression of *bla*_NDM−1_ of the last generation cells (OD600 reached 1.5-2.0 within 11–12 h) exposed to imipenem with grade increasing (4 µg/mL, 8 µg/mL, and 12 µg/mL), decreasing (12 µg/mL to 8 µg/mL, 8 µg/mL to 4 µg/mL, and 4 µg/mL to 0 µg/mL), and canceling doses (12 µg/mL to 0 µg/mL, 8 µg/mL to 0 µg/mL, and 4 µg/mL to 0 µg/mL).

The CDS sequence of *bla*_NDM− 1_ was obtained from the NCBI database to design its fluorescent quantitative primers (Fwd-5′-ACTGGATCAAGCAGGAGATCAACC-3′ and Rev-5′-CCATTGGC GGCGAAAGTCA-3′) with oligo 7.0. The mRNA was isolated from the strain exposed to imipenem by using TIANGEN RNAprep pure Cell/Bacteria Kit (China) and cDNA was synthesized by reverse transcription by using TOYOBO ReverTra Ace® qPCR RT Kit (Japan). The cDNA was used as the template to detect the expression levels of *bla*_NDM− 1_; the 16SrRNA gene was used as the reference gene. Results are presented as ratios of gene expression between the *bla*_NDM− 1_ (target) and the reference gene.

### Statistical analysis

GraphPadPrism 7 and SPSS 25.0 software were used for drawing and statistical analysis. All the experiments were repeated not less than 3 times, and the results were expressed by mean ± standard deviation. T-test was used to compare the data among groups, P-values of < 0.05 were considered statistically significant.

## Results

### Drug sensitivity analysis

The drug sensitivity results of clinical isolates showed that the carbapenem resistance rates of *K.pneumoniae* and *E.coli* were 18.6% (n = 19) and 1% (n = 1), respectively.

All randomly selected carbapenems-sensitive *K.pneumoniae* and *E.coli* strains showed resistance against selected antibiotics (Table 2), especially to cephalosporins, such as 35% (n = 7) and 75% (n = 15) of cefazolin (first generation), 40% (n = 8) and 70% (n = 14) of cefuroxime (second generation), 30% (n = 6) and 70% (n = 14) of ceftriaxone (third generation), 15% (n = 3) and 60% (n = 12) of cefepime (fourth generation), respectively. In addition, they also showed certain resistance to *β*-lactamase inhibitors, such as ampicillin/sulbactam of 30% (n = 6) and 35% (n = 7), and ticarcillin/clavulanic acid of 20% (n = 4) and 20% (n = 4), respectively. The results also showed that drug-resistant *K.pneumoniae* mostly came from sputum, while *E. coli* came from urine (Table S1). However, all strains of *K.pneumoniae* and *E.coli* were highly susceptible to carbapenems (MIC ˂1 µg/mL) (Table 2).

**Table 2:** Antimicrobial susceptibility patterns of carbapenem sensitive isolates against selected antibiotics

*Strains*	MIC(µg/mL) SAM	CAP	CIP	CRO	CFZ	CXM	FOX	CAZ	FEP	CN	TIM	PTZ	MI	SXT	LEV	IPM	MEM
*K.pneumoniae* (n = 20)	> 16/8 (n = 6)	≥ 32 (n = 2)	≥ 4 (n = 1)	≥ 64 (n = 6)	≥ 8 (n = 7)	≥ 32 (n = 8)	≥ 32 (n = 1)	≥ 32 (n = 5)	≥ 32 (n = 3)	≥ 16 (n = 2)	≥ 64 (n = 4)	≥ 32 (n = 1)	≥ 16 (n = 1)	≥ 8/152 (n **=** 5)	≥ 8 (n = 1)	˂1 (n = 20)	˂1 (n = 20)
*E.coli* (n = 20)	> 16/8 (n = 7)	≥ 32 (n = 1)	≥ 4 (n = 13)	≥ 64 (n = 14)	≥ 8 (n = 15)	≥ 32 (n = 14)	≥ 32 (n = 0)	≥ 32 (n = 6)	≥ 32 (n = 12)	≥ 16 (n = 7)	≥ 64 (n = 4)	≥ 32 (n = 10)	≥ 16 (n = 0)	≥ 8/152 (n = 15)	≥ 8 (n = 11)	˂1 (n = 20)	˂1 (n = 20)

SAM, Ampicillin/sulbactam; CAP, Chloramphenicol; CIP, Ciprofloxacin; CRO, Ceftriaxone; CFZ, Cefazolin; CXM, Cefuroxime; FOX, Cefoxitin; CAZ, Ceftazidime; FEP, Cefepime;

CN, Gentamicin; TIM, Ticarcillin/Clavulanic acid; PTZ, Piperacillin/tazobactam; MI, Minocycline; SXT, Sulfamethoxazole; LEV, Levofloxacin; IPM, Imipenem; MEM, Meropenem

### Molecular identification of ***β***-lactamase genes

**Fig. 1:**
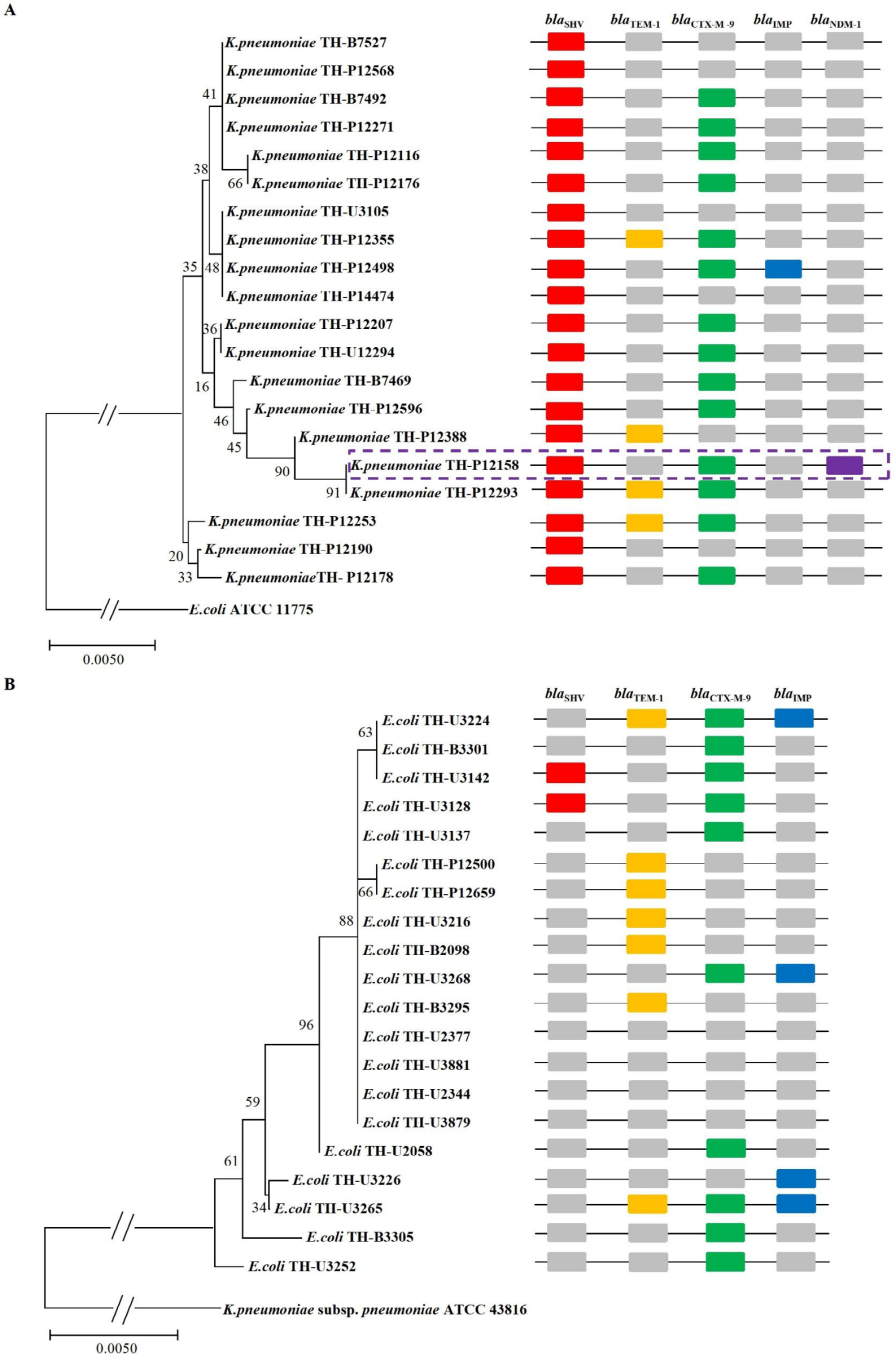
The *β*-lactamases genes carried by *K.pneumoniae* and *E.coli* strains. Based on the neighbor-joining method, the phylogenetic tree was constructed by comparing the 16SrDNA sequences of *K.pneumoniae* (A) and *E.coli* strains (B). The *β*-Lactamases genes of *bla*_SHV_ (red), *bla*_TEM−1_ (yellow), *bla*_CTX−M−9_ (green), *bla*_IMP_ (blue), and *bla*_NDM−1_ (purple) amplified by PCR were placed on the right side of the strain, but this did not indicate the location of these genes in the genome of the strain

The detected *β*-lactamase genes are shown in Fig. 1. The detection rates of *bla*_SHV_, *bla*_TEM−1_, *bla*_CTX−M−9_, and *bla*_IMP_ in *K.pneumoniae* were 100% (n = 20), 20% (n = 4), 70% (n = 14), and 5% (n = 1), respectively, while those in *E.coli* were 10% (n = 2), 35% (n = 7), 50% (n = 10), and 20% (n = 4) respectively. *K.pneumoniae* generally carried *bla*_SHV_ and *bla*_CTX−M−9_, while *E.coli* mostly carried *bla*_CTX−M−9_. Surprisingly, *bla*_NDM−1_ and *bla*_IMP_ (carbapenemase gene) were detected in *K.pneumoniae* (TH-P12158 and TH-P12498) (Fig. 1A) and *E.coli* (TH-U3224, TH-U3268, TH-U3226, and TH-U3265) (Fig. 1A, B).

### Imipenem exposure

**Fig. 2:**
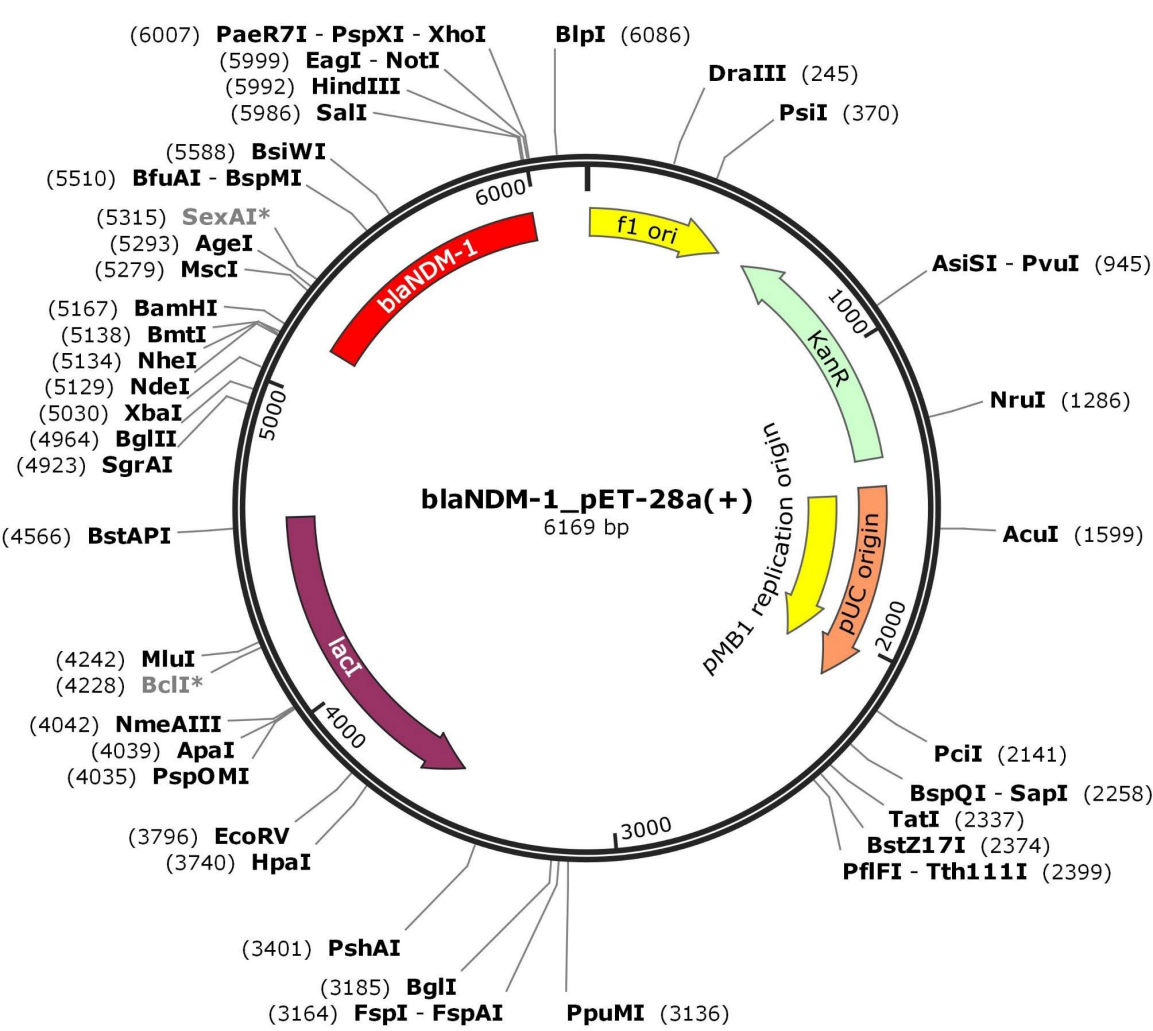
Structure of *bla*_NDM−1_-pET-28a (+) plasmid

The *bla*_NDM−1_ gene was inserted into BamH I and Sal I sites of the pET-28a (+) vector (Fig. S1, S2). The structure of *bla*_NDM−1_-pET28a (+) plasmid transferred to the competent cells of *E.coli* BL21 (DE3) and *E.coli* DH5α was displayed in Fig. 2.

### Subculture growth

**Fig. 3:**
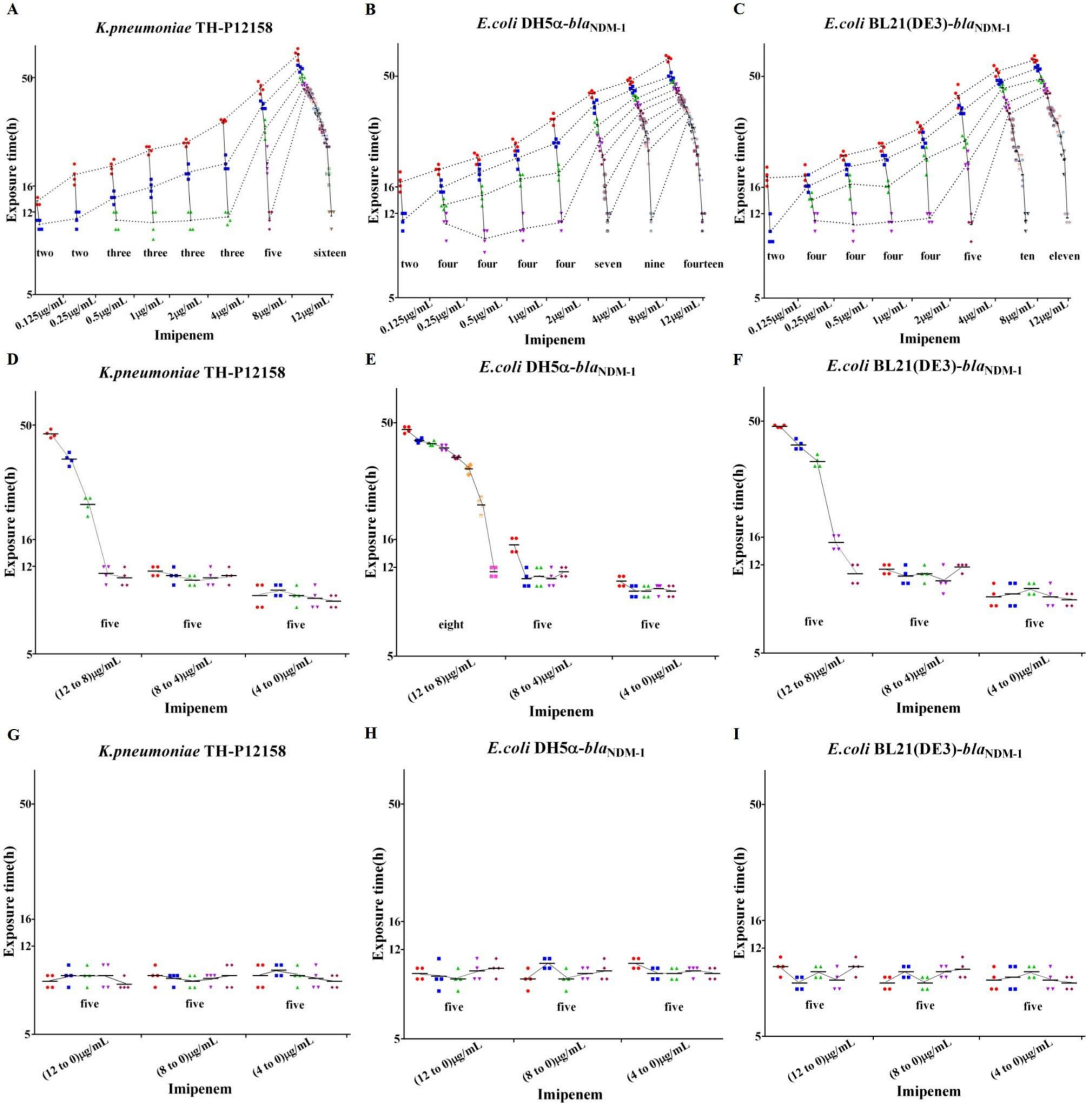
Effect of imipenem on the growth of all three strains. Firstly, the same strain was exposed to imipenem of 0.125 µg/mL (MIC ˂1 µg/mL), and then exposed to imipenem at grade increasing (0.25 µg/mL, 0.5 µg/mL, 1 µg/mL, 2 µg/mL, 4 µg/mL, 8 µg/mL, and 12 µg/mL), decreasing (12 µg/mL to 8 µg/mL, 8 µg/mL to 4 µg/mL, and 4 µg/mL to 0 µg/mL), and canceling (12 µg/mL to 0 µg/mL, 8 µg/mL to 0 µg/mL, and 4 µg/mL to 0 µg/mL) doses, respectively. Bacterial growth curve was drawn with GraphPad 7.0 software

Effects of imipenem on bacterial growth are displayed in Fig. 3. The MICs of imipenem against *K.pneumoniae* TH-P12158, *E.coli* BL21 (DE3)-*bla*_NDM−1_, and *E.coli* DH5α-*bla*_NDM−1_ were 0.5 µg/mL, 0.25 µg/mL, and 0.25 µg/mL, respectively. With the increased doses of imipenem, the subculture times of *K.pneumoniae*TH-P12158, *E.coli* BL21 (DE3)-*bla*_NDM−1_, and *E.coli* DH5α-*bla*_NDM−1_ were also increased. However, *E.coli* BL21 (DE3)-*bla*_NDM−1_ and *E.coli*DH5α-*bla*_NDM−1_ need more subculture times to make them grow normally (11-12 h, OD_600_ = 1.5-2.0) than *K.pneumoniae* TH-P12158 (Fig. 3A, B, C).

From Fig. 3, it can be seen that the subculture times of three strains were significantly shortened at the decreased doses of imipenem (12 µg/mL to 8 µg/mL, 8 µg/mL to 4 µg/mL, 4 µg/mL to 0 µg/mL) (Fig. 3D, E, and F). When the imipenem was canceled, the cells of the same strain exposed to imipenem of 12 µg/mL, 8 µg/mL, or 4 µg/mL could proliferate to OD_600_ of 1.5-2.0 in 8-12 h with the same subculture times (Fig. 3G, H, and I).

### MIC, MBC, and K-B method

**Table 3:** The MIC and MBC values of seven antimicrobial agents against three strains exposed to imipenem

Strains	imipenem(µg/mL) MIC(µg/mL)/MBC(µg/mL)							
		IPM	MEM	CXM	CAZ	CSSS	PSTS	LEV
*K.pneumoniae* TH-P12158	0	0.5/1	0.5/1	8/32	2/8	8/32	8/32	0.125/0.25
	4	4/8	1/4	8/32	2/8	8/32	8/32	0.125/0.25
	8	8/16	1/4	16/64	2/8	8/32	16/64	0.125/0.25
	12	8/16	2/8	16/64	4/16	8/32	16/64	0.125/0.25
	12 to 8	8/16	2/8	16/64	4/16	8/32	16/64	0.125/0.25
	8 to 4	8/16	2/8	16/64	4/16	8/32	16/64	0.125/0.25
	4 to 0	8/16	2/8	16/64	4/16	8/32	16/64	0.125/0.25
	12 to 0	8/16	2/8	16/64	4/16	8/32	16/64	0.125/0.25
	8 to 0	8/16	2/8	16/64	4/16	8/32	16/64	0.125/0.25
	4 to 0	8/16	2/8	16/64	4/16	8/32	16/64	0.125/0.25
*E.coli* DH5α-*bla*_NDM−1_	0	0.25/1	0.25/0.5	8/32	2/8	1/2	8/8	0.125/0.25
	4	2/4	0.5/1	16/32	2/8	4/16	16/16	0.125/0.25
	8	4/8	0.5/1	16/32	2/8	4/16	16/16	0.125/0.25
	12	4/8	0.5/1	16/32	4/16	4/16	16/16	0.125/0.25
	12 to 8	4/8	0.5/1	16/32	4/16	2/8	16/16	0.125/0.25
	8 to 4	4/8	0.5/1	16/32	4/16	2/8	16/16	0.125/0.25
	4 to 0	4/8	0.5/1	16/32	4/16	2/8	16/16	0.125/0.25
	12 to 0	4/8	0.5/1	16/32	4/16	2/8	16/16	0.125/0.25
	8 to 0	4/8	0.5/1	16/32	4/16	2/8	16/16	0.125/0.25
	4 to 0	4/8	0.5/1	16/32	4/16	2/8	16/16	0.125/0.25
*E.coli*BL21(DE3)-*bla*_NDM−1_	0	0.25/1	0.25/0.5	8/64	4/128	2/8	2/8	0.125/0.25
	4	2/4	0.5/1	8/64	4/128	4/16	4/16	0.125/0.25
	8	4/8	0.5/1	8/64	4/128	4/16	4/16	0.125/0.25
	12	4/8	0.5/1	16/64	8/128	4/16	4/16	0.125/0.25
	12 to 8	4/8	0.5/1	16/64	8/128	4/16	4/16	0.125/0.25
	8 to 4	4/8	0.5/1	16/64	8/128	4/16	4/16	0.125/0.25
	4 to 0	4/8	0.5/1	16/64	8/128	4/16	4/16	0.125/0.25
	12 to 0	4/8	0.5/1	16/64	8/128	4/16	4/16	0.125/0.25
	8 to 0	4/8	0.5/1	16/64	8/128	4/16	4/16	0.125/0.25
	4 to 0	4/8	0.5/1	16/64	8/128	4/16	4/16	0.125/0.25

IPM, imipenem; MEM, meropenem; CXM, cefuroxime; CAZ, ceftazidime; CSSS, cefoperazone sodium/sulbactam sodium; PSTS, piperacillin sodium /tazobactam sodium; LEV, levofloxacin

The MIC values of seven antimicrobial agents against three strains exposed to imipenem are displayed in Table 3. Under the exposure of imipenem of 0 µg/mL, 4 µg/mL, 8 µg/mL, and 12 µg/mL, the MIC values of Imipenem against *K.pneumoniae* TH-12,158, *E.coli* BL21(DE3)-*bla*_NDM−1_, and *E.coli* DH5α-*bla*_NDM−1_ were increased from 1×MIC (0.5 µg/mL), 1×MIC (0.25 µg/mL), and 1×MIC (0.25 µg/mL) at 0 µg/mL to 16×MIC, 8×MIC,and 8×MIC at 12 µg/mL, respectively. Meanwhile, the MIC values of meropenem and other drugs except norfloxacin were increased significantly. When the doses of imipenem were decreased or canceled from high to low, the MIC values of imipenem against the three strains remained unchanged for 20 generations (Table S2) (subsequent experiments were not done).

The same antibacterial effect also showed that the MBC values of seven antimicrobial agents were 2–4 times that of MIC, whether the exposure doses increased or decreased, or canceled on the strains (Table 3).

**Table 4:** Detection of drug resistance of three strains exposed to imipenem using the K-B method

Imipenem (µg/mL)	Inhibition zone (mm)		
	*K.pneumoniae* TH-P12158	*E.coli* DH5α-*bla*_NDM−1_	*E.coli* BL21(DE3)-*bla*_NDM−1_
0	26.00 ± 2.45	30.00 ± 2.16	26.00 ± 1.63
4	16.33 ± 1.25	17.67 ± 0.47	16.67 ± 0.94
8	13.67 ± 0.47	14.67 ± 0.47	14.33 ± 0.47
12	11.00 ± 0.00	12.67 ± 0.47	13.00 ± 0.82
12 to 8	9.67 ± 0.94	12.33 ± 0.47	13.33 ± 0.47
8 to 4	9.00 ± 1.63	11.00 ± 0.00	11.67 ± 0.94
4 to 0	9.00 ± 0.82	11.00 ± 0.00	12.33 ± 0.47
12 to 0	10.33 ± 0.47	12.67 ± 0.47	13.33 ± 0.94
8 to 0	9.00 ± 1.63	11.33 ± 0.47	12.33 ± 1.25
4 to 0	9.00 ± 0.82	11.00 ± 0.00	12.33 ± 0.47

The resistance changes of three strains exposed to imipenem at series doses were shown in Table 4. Under the exposure of imipenem of 0 µg/mL, 4 µg/mL, 8 µg/mL, and 12 µg/mL, the inhibition zone of imipenem against *K.pneumoniae* TH-P12158 was significantly reduced from 26.00 ± 2.45 mm to 16.33 ± 1.25 mm (p < 0.05), 13.67 ± 0.47 mm (p < 0.05), and 11.00 ± 0.00 mm (p < 0.05), respectively. Meanwhile, *E.coli* DH5α-*bla*_NDM−1_ and *E.coli* BL21 (DE3)-*bla*_NDM−1_ showed also gradually increasing resistance to imipenem from 30.00 ± 2.16 mm, and 26.00 ± 1.63 mm of 0 µg/mL to 12.67 ± 0.47 mm (p < 0.05) and 13.00 ± 0.82 mm (p < 0.05) of 12 µg/mL, respectively.

Table 4 also showed that the inhibition zone of all strains exposed to imipenem of 12 µg/ml did not change significantly, regardless of whether the imipenem was decreased or canceled.

### qRT-PCR

**Fig. 4:**
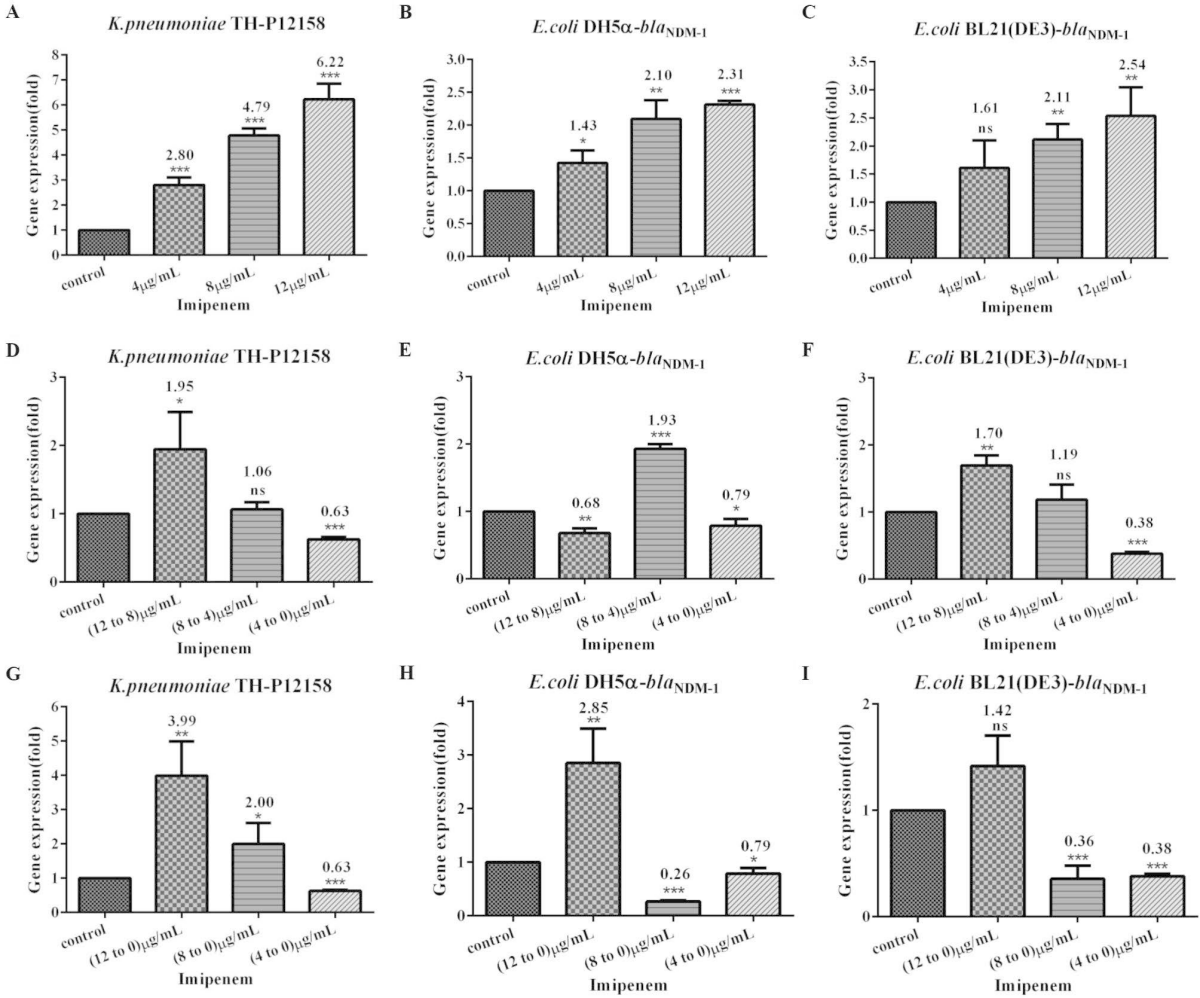
Effect of imipenem on the *bla*_NDM−1_ expression of all three strains. The qRT-PCR was applied to detect the expression of *bla*_NDM−1_ of the last generation cells (OD_600_ reached 1.5-2.0 within 11-13 h) exposed to imipenem with grade increasing (control, 4 µg/mL, 8 µg/mL, and 12 µg/mL), decreasing (12 µg/mL to 8 µg/mL, 8 µg/mL to 4 µg/mL, and 4 µg/mL to 0 µg/mL), and canceling (12 µg/mL to 0 µg/mL, 8 µg/mL to 0 µg/mL, and 4 µg/mL to 0 µg/mL) doses, respectively

The expression levels of *bla*_NDM−1_ of all three strains exposed to imipenem were shown in Fig. 4. Under the exposure of imipenem of 0 µg/mL (control), 4 µg/mL, 8 µg/mL, and 12 µg/mL, the expression values of *bla*_NDM−1_ of *K.pneumoniae*TH-P12158 were approximately threefold (relative quantification [RQ] = 2.8, p < 0.001 ), fivefold (RQ = 4.79, p < 0.001), and sevenfold (RQ = 6.22, p < 0.001) compared to the control without imipenem, respectively (Fig. 4A). Based on the same method, the expression levels of *bla*_NDM−1_ of *E.coli* DH5α-*bla*_NDM−1_ and *E.coli* BL21 (DE3)-*bla*_NDM−1_ were found to be lower than *K.pneumoniae* TH-P12158. The expression values of *bla*_NDM−1_ of *E.coli* DH5α-*bla*_NDM−1_ were approximately twofold (RQ = 1.43, p < 0.05), twofold (RQ = 2.10, p < 0.01), and twofold (RQ = 2.31, p < 0.001) from the control (RQ = 1), respectively (Fig. 4B). Meanwhile, the RQ expression values of *bla*_NDM−1_ of *E.coli* BL21 (DE3)-*bla*_NDM−1_ also were up-regulated 1.61(p > 0.05), 2.11(p < 0.01), and 2.54(p < 0.01) (Fig. 4C). With the gradually decreased or canceled exposure to imipenem, the expression levels of *bla*_NDM−1_ of all three strains were gradually decreased, and the RQ value was even less than 0.4 (Fig. 4F, H, I). However, the expression values of strains exposed to imipenem at 12 µg/mL to 8 µg/mL or 12 µg/mL to 0 µg/mL were higher than the control, suggesting that the strain exposed to imipenem of 12 µg/mL produced relatively stable *bla*_NDM−1_ expression (Fig. 4G, H, I), and the MIC values also supported this speculation.

## Discussion

*K.pneumoniae* and *E.coli* are important pathogens of community- and hospital-acquired infections, and they are also multi-drug-resistant bacteria posing a serious threat to the clinic. *K.pneumoniae* and *E.coli* generally carry many types of *β*-lactamase genes, such as early common *bla*_TEM_, *bla*_SHV_, and *bla*_CTX−M_, *bla*_KPC_, and *bla*_NDM−1_, which have become mainstream in recent years [13, 26–29]. The coexistence of *bla*_NDM−1_ and other *β*-lactamase genes, and their drug resistance phenotype have attracted much attention; however, not enough attention was drawn to the sensitive bacteria carrying *bla*_NDM−1_. In this study, the gene analysis of 40 isolates showed that the higher cephalosporin resistance rates of *K.pneumoniae* and *E.coli* were probably related to their drug resistance genes. *K.pneumoniae* generally carried *bla*_SHV_ (100%, n = 20) and *bla*_CTX−M−9_ (70%, n = 14). *K.pneumoniae* TH-P12498 and *K.pneumoniae* TH-P12158 carried *bla*_IMP_ and *bla*_NDM−1_ of carbapenemase genes respectively and coexisted with *bla*_SHV_ and *bla*_CTX−M−9_. Compared with *K.pneumoniae, E.coli* carried higher *bla*_TEM−1_ (35%, n = 7) and *bla*_CTX−M−9_ (50%, n = 10). Among them, all four strains of *E.coli* (TH-U3224, TH-U3226, TH-U3265, and TH-U3268) isolated from patients’ urine samples carried carbapenem gene *bla*_IMP_ and coexisted with *bla*_TEM−1_ and *bla*_CTX−M−9_. The phenotypic and genotypic characteristics of 40 isolates showed that *bla*_SHV_, *bla*_TEM−1_, *bla*_CTX−M−9_, and *bla*_IMP_ might be transmitted horizontally within or between *K.pneumoniae* and *E.coli*. The high carrier rate of *bla*_IMP_ in *E.coli* should be highlighted, although strains did not show carbapenem resistance phenotype.

The rapid expansion of acquired carbapenem resistance is increasingly propagated by mobile genetic elements such as epidemic plasmids that transfer carbapenemase genes within and between *Enterobacteriaceae* [27]. Plasmids harboring *bla*_NDM_ are frequently larger than 100 kb and belong to the incompatibility groups IncA/C, IncX3 or IncF with a broad-host range. The higher fitness cost of *bla*_NDM−1_ plasmid in *E.coli* may determine that it mostly exists in *K.pneumoniae*, but rarely in *E.coli* [26]. Although our study also found that most of the *bla*_NDM−1_ positive drug-resistant bacteria are *K.pneumoniae*, the wild plasmid (123KB) coexisting with other drug-resistant genes (*bla*_CTX−M−9_, *bla*_TEM−1_), or the artificially constructed *bla*_NDM−1_-pET28a (+) plasmid can be transferred to *E. coli*[14].

Wild type or artificially constructed *bla*_NDM−1_ plasmid do not show carbapenem resistance phenotype (MIC ˂1 µg/mL), which may be related to a variety of factors, such as host compatibility, antibiotic stress, and so on. Antibiotics are considered to be the most important driving force for accelerating the transformation of drug-resistant genes. They exert selective pressure on bacteria to make the mutation of chromosome genes and the spread of existing or rising genes, thus enhancing the resistance and virulence of bacteria [11, 12, 27, 30]. Antibiotic-induced mutagenesis is typically studied at antibiotic concentrations close to, but below the MIC value [28]. Under the exposure of imipenem, meropenem, and ertapenem stress, an upregulated expression of *bla*_NDM−1_ was observed by quantitative real-time polymerase chain reaction [18]. *K.pneumoniae* TH-P12158, *E.coli* DH5α-*bla*_NDM−1_, and *E.coli* BL21 (DE3)-*bla*_NDM−1_ showed resistance phenotype and genotype changes positively correlated with increased imipenem exposure, this suggests that the *bla*_NDM−1_ experienced the same up-regulation. However, with the decrease or cancellation of imipenem exposure, the expression of *bla*_NDM−1_ was down-regulated, although the resistant phenotype remained unchanged. Based on the MIC and MBC values of imipenem, we can confirm that the resistant phenotypes of strains carrying *bla*_NDM−1_ have produced stable memory.

Cross-resistance is a phenomenon where the acquisition of resistance to a specific drug causes resistance to another drug simultaneously [29]. *K.pneumoniae* TH-P12158, *E.coli* DH5α-*bla*_NDM−1_, and *E.coli* BL21 (DE3)-*bla*_NDM−1_ showed enhanced resistance to exposed and unexposed antibiotics (MEM, CXM, CAZ, CSS, and PSTS), indicating that a specific antibiotic exposure will inevitably lead to an expanded antimicrobial spectrum. We do not know whether imipenem can specifically press *bla*_NDM−1_ or only is an antibiotic stress factor. But so far, it is certain that the drug resistance phenotype of the exposed strains can be stably sub-cultured with chromosome mutation and horizontal transmission to the 20th passage (Table S3). Special attention should be paid to drug resistance caused by the mutation of sensitive bacteria or the horizontal transfer of drug-resistant genes caused by antibiotic exposure. The transmission or expression of drug-resistant genes after the decrease or cancellation of exposure factors is more significant for the use of clinical drugs.

## Electronic supplementary material

Below is the link to the electronic supplementary material.

Additional files 1: Table S1. The resistance of 20 clinical isolates to 17 antibiotics.
  Additional files 2: Table S2. Transmission of drug resistance phenotype of imipenem exposed strains in subculture cells.
  Additional files 3: Fig. S1. The electropherogram map of *bla*_NDM−1_-pET28a (+) plasmid.
  Additional files 4: Fig. S2. The electropherogram map of *bla*_NDM−1_ gene amplified from *bla*_NDM−1_-pET28a (+) plasmid.

## Data Availability

The datasets used or analysed during the current study are available from the corresponding author on reasonable request.

## Abbreviations

*bla*_NDM−1_: New Delhi Metallo-β-Lactamase-1 gene
MIC: Minimum inhibitory concentration
MBC: Minimum bactericidal concentration
DRE: Drug-resistant *Enterobacteriaceae*
ESBLs: Extended-spectrum *β*-lactamases
MBLs: Metallo-*β*-Lactamases
NDM-1: New Delhi Metallo-β-Lactamases-1
qRT-PCR: Quantitative real-time-PCR
SAM: Ampicillin/sulbactam
CAP: Chloramphenicol
CIP: Ciprofloxacin
CRO: Ceftriaxone
CFZ: Cefazolin
CXM: Cefuroxime
FOX: Cefoxitin
CAZ: Ceftazidime
FEP: Cefepime
CN: Gentamicin
TIM: Ticarcillin/Clavulanic acid
PTZ: Piperacillin/tazobactam
MI: Minocycline
SXT: Sulfamethoxazole
LEV: Levofloxacin
IPM: Imipenem
MEM: Meropenem
CSSS: Cefoperazone sodium/sulbactam sodium
PSTS: Piperacillin sodium /tazobactam sodium.
