# Supplementary material for: Evolution of carbapenem resistance in klebsiella pneumoniae and escherichia coli carrying blaNDM−1 gene: imipenem exposure results in sustained resistance memory of strains in vitro
Source: Ann Clin Microbiol Antimicrob. 2023 Jun 12;22:46. doi: 10.1186/s12941-023-00598-8 (PMC10262507; doi:10.1186/s12941-023-00598-8)
Supplement: Supplementary file 1 — Additional files 1: Table S1. The resistance of 20 clinical isolates to 17 antibiotics. Additional files 2: Table S2. Transmission of drug resistance phenotype of imipenem exposed strains in subculture cells. Additional files 3: Fig. S1. The electropherogram map of blaNDM−1-pET28a (+) plasmid. Additional files 4: Fig. S2. The electropherogram map of blaNDM−1 gene amplified from blaNDM−1-pET28a (+) plasmid. [file 12941_2023_598_MOESM1_ESM.doc]

**Evolution of carbapenem resistance in** ***Klebsiella pneumoniae* and *Escherichia coli* carrying *bla*_NDM-1_ gene: imipenem exposure results in sustained resistance memory of strains in vitro**

Qiong Zhao^1^, Longhua Sha^1^, Zhaomen Wu^1^, Lixue Meng^1^, Feixiang Yang^2^, Lingling Wu^2^, Chunfang Yu^1^, Hua Zhang ^2*^, Jindan Yu^3*^, Zhixiong Jin^1, 2, 4*^

^1^Department of Microbiology, School of Basic Medical Sciences, Hubei University of Medicine, Hubei, Shiyan 442000, China

^2^ Department of Clinical Laboratory, Sinopharm Dongfeng General Hospital, Hubei University of Medicine, Hubei, Shiyan, 442008, China

^3^Laboratory Medicine, Wuhan Asia General Hospital, Hubei, Wuhan 430050, China

^4^Hubei Key Laboratory of Wudang Local Chinese Medicine Research, Hubei University of Medicine, Hubei, Shiyan 442000, China

*Corresponding author.

*E-mail address:* jzx8801150@126.com (Z.X.Jin)

**Supplementary information**

Additional files 1: Table S1. The resistance of 40 clinical isolates to 17 antibiotics.

Additional files 2: Table S2. Transmission of drug resistance phenotype of imipenem exposed strains in subculture cells.

Additional files 3: Fig. S1. The electropherogram map of *bla*_NDM-1_-pET28a (+) plasmid.

Additional files 4: Fig. S2. The electropherogram map of *bla*_NDM-1_ gene amplified from *bla*_NDM-1_-pET28a (+) plasmid.

**Table S1** The resistance of 40 clinical isolates to 17 antibiotics

| Strains sample MIC(µg/mL) | | | | | | | | | | | | | | | | | | |
| --- | --- | --- | --- | --- | --- | --- | --- | --- | --- | --- | --- | --- | --- | --- | --- | --- | --- | --- |
|  |  | SAM | CAP | CIP | CRO | CFZ | CXM | FOX | CAZ | FEP | CN | TIM | PTZ | MI | SXT | LEV | IPM | MEM |
| *K.pneumoniae*  (n=20) | Sputum (n=11) | >16/8  (n=4) | ≥32  (n=2) | ≥4  ( n=1) | ≥64  (n=3) | ≥8 (n=4) | ≥32  (n=5) | ≥32  (n=0) | ≥32  (n=4) | ≥32  (n=2) | ≥16  (n=1) | ≥64  (n=3) | ≥32 (n=1) | ≥16  (n=1) | ≥8/152 (n=1) | ≥8  (n=1) | ≥4  (n=0) | ≥4 (n=0) |
|  | Blood  (n=3) | >16/8  (n=1) | ≥32  (n=0) | ≥4  (n=0) | ≥64 (n=1) | ≥8  (n=1) | ≥32  (n=1) | ≥32  (n=1) | ≥32  (n=1) | ≥32  (n=1) | ≥16  (n=1) | ≥64  (n=1) | ≥32  (n=0) | ≥16  (n=0) | ≥8/152 (n=1) | ≥8  (n=0) | ≥4  (n=0) | ≥4 (n=0) |
|  | Urine  (n=1) | >16/8  (n=0) | ≥32  (n=0) | ≥4  (n=0) | ≥64 (n=0) | ≥8  (n=0) | ≥32 (n=0) | ≥32  (n=0) | ≥32  (n=0) | ≥32  (n=0) | ≥16  (n=0) | ≥64  (n=0) | ≥32  (n=0) | ≥16  (n=0) | ≥8/152 (n=1) | ≥8  (n=0) | ≥4 (n=0) | ≥4 (n=0) |
|  | Others  (n=5) | >16/8  (n=1) | ≥32  (n=0) | ≥4  (n=0) | ≥64  (n=2) | ≥8  (n=2) | ≥32  (n=2) | ≥32  (n=0) | ≥32  (n=0) | ≥32  (n=0) | ≥16  (n=0) | ≥64  (n=0) | ≥32  (n=0) | ≥16  (n=0) | ≥8/152 (n=2) | ≥8  (n=0) | ≥4 (n=0) | ≥4 (n=0) |
| *E.coli*  (n=20) | Sputum (n=2) | >16/8  (n=1) | ≥32 (n=0) | ≥4  (n=0) | ≥64  (n=2) | ≥8  (n=2) | ≥32  (n=2) | ≥32  (n=0) | ≥32  (n=2) | ≥32  (n=2) | ≥16  (n=2) | ≥64  (n=1) | ≥32  (n=0) | ≥16  (n=0) | ≥8/152 (n=2) | ≥8  (n=0) | ≥4 (n=0) | ≥4 (n=0) |
|  | Blood  (n=3) | >16/8  (n=2) | ≥32 (n=0) | ≥4  (n=3) | ≥64  (n=2) | ≥8  (n=2) | ≥32  (n=2) | ≥32  (n=0) | ≥32  (n=1) | ≥32  (n=2) | ≥16  (n=0) | ≥64  (n=1) | ≥32  (n=0) | ≥16  (n=0) | ≥8/152 (n=0) | ≥8  (n=3) | ≥4 (n=0) | ≥4 (n=0) |
|  | Urine  (n=11) | >16/8  (n=3) | ≥32 (n=0) | ≥4  (n=7) | ≥64  (n=8) | ≥8  (n=9) | ≥32  (n=8) | ≥32  (n=0) | ≥32  (n=2) | ≥32  (n=6) | ≥16  (n=3) | ≥64  (n=1) | ≥32  (n=1) | ≥16  (n=0) | ≥8/152 (n=10) | ≥8  (n=5) | ≥4 (n=0) | ≥4 (n=0) |
|  | Others (n=4) | >16/8  (n=1) | ≥32 (n=1) | ≥4  (n=3) | ≥64  (n=2) | ≥8  (n=2) | ≥32  (n=2) | ≥32  (n=0) | ≥32  (n=1) | ≥32  (n=2) | ≥16  (n=2) | ≥64  (n=1) | ≥32  (n=0) | ≥16  (n=0) | ≥8/152 (n=3) | ≥8  (n=3) | ≥4 (n=0) | ≥4 (n=0) |

n, number; SAM, Ampicillin/sulbactam; CAP, Chloramphenicol; CIP, Ciprofloxacin; CRO, Ceftriaxone; CFZ, Cefazolin; CXM, Cefuroxime; FOX, Cefoxitin; CAZ, Ceftazidime; FEP, Cefepime; CN, Gentamicin; TIM, Ticarcillin/Clavulanic acid; PTZ, Piperacillin/tazobactam; MI, Minocycline; SXT, Sulfamethoxazole; LEV, Levofloxacin; IPM, Imipenem; MEM, Meropenem.

**Table S2** Transmission of drug resistance phenotype of imipenem exposed strains in subculture cells.

| Strains | Imipenem(µg/ml) | MIC(µg/mL) |  |  |  |  |  |  |  |  |  |  |  |  |  |  |  |  |  |  |  |
| --- | --- | --- | --- | --- | --- | --- | --- | --- | --- | --- | --- | --- | --- | --- | --- | --- | --- | --- | --- | --- | --- |
|  |  | 1 | 2 | 3 | 4 | 5 | 6 | 7 | 8 | 9 | 10 | 11 | 12 | 13 | 14 | 15 | 16 | 17 | 18 | 19 | 20 |
| *K.pneumoniae*TH-P12158  *E.coli* DH5α-*bla*_NDM-1_  *E.coli*BL21(DE3)-*bla*_NDM-1_ | 12 to 0 | 8 | 8 | 8 | 8 | 8 | 8 | 8 | 8 | 8 | 8 | 8 | 8 | 8 | 8 | 8 | 8 | 8 | 8 | 8 | 8 |
|  | 8 to 0 | 8 | 8 | 8 | 8 | 8 | 8 | 8 | 8 | 8 | 8 | 8 | 8 | 8 | 8 | 8 | 8 | 8 | 8 | 8 | 8 |
|  | 4 to 0 | 8 | 8 | 8 | 8 | 8 | 8 | 8 | 8 | 8 | 8 | 8 | 8 | 8 | 8 | 8 | 8 | 8 | 8 | 8 | 8 |
|  | 12 to 0 | 4 | 4 | 4 | 4 | 4 | 4 | 4 | 4 | 4 | 4 | 4 | 4 | 4 | 4 | 4 | 4 | 4 | 4 | 4 | 4 |
|  | 8 to 0 | 4 | 4 | 4 | 4 | 4 | 4 | 4 | 4 | 4 | 4 | 4 | 4 | 4 | 4 | 4 | 4 | 4 | 4 | 4 | 4 |
|  | 4 to 0 | 4 | 4 | 4 | 4 | 4 | 4 | 4 | 4 | 4 | 4 | 4 | 4 | 4 | 4 | 4 | 4 | 4 | 4 | 4 | 4 |
|  | 12 to 0 | 4 | 4 | 4 | 4 | 4 | 4 | 4 | 4 | 4 | 4 | 4 | 4 | 4 | 4 | 4 | 4 | 4 | 4 | 4 | 4 |
|  | 8 to 0 | 4 | 4 | 4 | 4 | 4 | 4 | 4 | 4 | 4 | 4 | 4 | 4 | 4 | 4 | 4 | 4 | 4 | 4 | 4 | 4 |
|  | 4 to 0 | 4 | 4 | 4 | 4 | 4 | 4 | 4 | 4 | 4 | 4 | 4 | 4 | 4 | 4 | 4 | 4 | 4 | 4 | 4 | 4 |

Taking OD_600_ of 1.5-2.0 (1.5×10^8^ CFU/mL) as the subculture growth standard of strains with 11-12h, the strains exposed for imipenem of 4µg/mL, 8µg/mL, and 12µg/mL were subcultured in liquid medium without any antibiotics for 20 generations to detect the MIC value of imipenem.


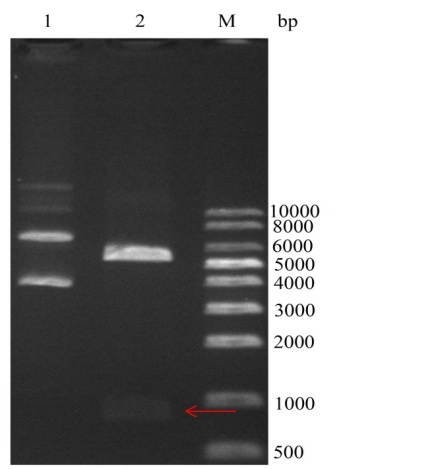


**Fig.S1** The electropherogram map of *bla*_NDM-1_-pET28a (+) plasmid.

M, marker; lane 1, the gene of *bla*_NDM-1_-pET28a (+) plasmid; lane 2, the *bla*_NDM-1_gene of *bla*_NDM-1_-pET28a (+) plasmid digested with BamHI & SalI (red arrow).


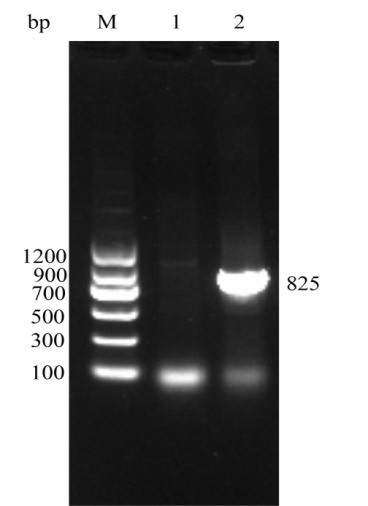


**Fig.S2** The electropherogram map of *bla*_NDM-1_ gene amplified from *bla*_NDM-1_-pET28a (+) plasmid.

M, DNA marker; lane 1, the negative control of *bla*_NDM-1_ gene; lane 2, the *bla*_NDM-1_ gene amplified from *bla*_NDM-1_-pET28a (+) plasmid.
